# Functional Connectivity Density Mapping of Depressive Symptoms and Loneliness in Non-Demented Elderly Male

**DOI:** 10.3389/fnagi.2015.00251

**Published:** 2016-01-12

**Authors:** Chen-Chia Lan, Shih-Jen Tsai, Chu-Chung Huang, Ying-Hsiu Wang, Tong-Ru Chen, Heng-Liang Yeh, Mu-En Liu, Ching-Po Lin, Albert C. Yang

**Affiliations:** ^1^Department of Psychiatry, Taichung Veterans General HospitalTaichung, Taiwan; ^2^Institute of Brain Science, National Yang-Ming UniversityTaipei, Taiwan; ^3^Department of Psychiatry, Taipei Veterans General HospitalTaipei, Taiwan; ^4^Division of Psychiatry, School of Medicine, National Yang-Ming UniversityTaipei, Taiwan; ^5^Taipei Veterans HomeTaipei, Taiwan; ^6^Center for Dynamical Biomarkers and Translational Medicine, National Central UniversityChungli, Taiwan; ^7^Division of Interdisciplinary Medicine and Biotechnology, Beth Israel Deaconess Medical Center/Harvard Medical SchoolBoston, MA, USA

**Keywords:** brain imaging, depressive symptoms, loneliness, functional connectivity density, elderly

## Abstract

**Background:** Depression and loneliness are prevalent and highly correlated phenomena among the elderly and influence both physical and mental health. Brain functional connectivity changes associated with depressive symptoms and loneliness are not fully understood.

**Methods:** A cross-sectional functional MRI study was conducted among 85 non-demented male elders. Geriatric depression scale-short form (GDS) and loneliness scale were used to evaluate the severity of depressive symptoms and loneliness, respectively. Whole brain voxel-wise resting-state functional connectivity density (FCD) mapping was performed to delineate short-range FCD (SFCD) and long-range FCD (LFCD). Regional correlations between depressive symptoms or loneliness and SFCD or LFCD were examined using general linear model (GLM), with age incorporated as a covariate and depressive symptoms and loneliness as predictors.

**Results:** Positive correlations between depressive symptoms and LFCD were observed in left rectal gyrus, left superior frontal gyrus, right supraorbital gyrus, and left inferior temporal gyrus. Positive correlations between depressive symptoms and SFCD were observed in left middle frontal gyrus, left superior frontal gyrus, bilateral superior medial frontal gyrus, left inferior temporal gyrus, and left middle occipital region. Positive correlations between SFCD and loneliness were centered over bilateral lingual gyrus.

**Conclusion:** Depressive symptoms are associated with FCD changes over frontal and temporal regions, which may involve the cognitive control, affective regulation, and default mode networks. Loneliness is associated with FCD changes in bilateral lingual gyri that are known to be important in social cognition. Depressive symptoms and loneliness may be associated with different brain regions in non-demented elderly male.

## Introduction

Depression and loneliness are both prevalent phenomena among elderly populations. A cross-sectional study with 359 subjects aged 74 years and older revealed the overall prevalence of depression was 25.1% and an additional 5.6% of the subjects exhibited subthreshold depressive symptoms (Forlani et al., 2014). Another population-based study in the United States with 10,409 subjects aged 55 years and older demonstrated that 13.7% had major depressive disorder (MDD) and 13.8% had subsyndromal depression (Laborde-Lahoz et al., 2015). As a social species, humans experience loneliness caused by impaired social relationships. The exact definition of loneliness varies between cultures or disciplines. However, the most widely accepted definition of loneliness is a subjective negative feeling associated with a perceived insufficiency of social network (social loneliness) or a lack of desired social companion (emotional loneliness; Valtorta and Hanratty, 2012). Around 2−28% of adults aged 65 or older reported severe loneliness (Wenger et al., 1996; Luo and Waite, 2014). The prevalence rate of loneliness was even higher among institutionalized older adults; in a study focusing on nursing home residents without cognitive impairment, more than half of them reported feeling lonely (Drageset et al., 2011).

Associations between depressive symptoms and loneliness have been repeatedly demonstrated. In a cross-sectional study with 6659 participants aged 65–80 randomly sampled from the total population in Sweden, an association between the odds to have a depressive disorder and loneliness was found (Djukanović et al., 2015). Another study conducted among 249 community-dwelling older adults in the Netherlands also demonstrated that loneliness was highly prevalent among those with depressive symptoms and that lonely people suffered from more severe depressive symptoms and higher risk of depressive disorders (van Beljouw et al., 2014).

Depression has multiple deleterious effects on mental well-being and also on physical health. Previous studies have demonstrated its negative impacts on patients with physical diseases, including poor quality of life, more functional impairments, and higher mortality rates (Kang et al., 2015). Of note, depressive symptoms have also been associated with similar negative influences including reduced quality of life, greater disability, increased physical morbidity and mortality (Cherubini et al., 2012). Similarly, loneliness also exerts detrimental effects on both physical and mental health. Loneliness has been demonstrated to be a risk factor or poor prognosticator for various physical conditions, such as hypertension (Momtaz et al., 2012), coronary heart disease (Thurston and Kubzansky, 2009), obesity and metabolic syndrome (Whisman, 2010), and altered immune stress response (Jaremka et al., 2013). Loneliness has also been associated with increased rates of all-cause mortality in elderly population (Tilvis et al., 2011). In addition, loneliness has been linked to increased risk of other mental problems, including anxiety (Ribeiro et al., 2015), alcohol abuse (Schonfeld and Dupree, 1991), insomnia (Segrin and Burke, 2015), dementia (Holwerda et al., 2014), and suicide (Stravynski and Boyer, 2001).

Despite the increasing number of studies focusing on the impact of depression and loneliness, brain alterations associated with depressive symptoms or loneliness in elderly population are still poorly understood. Depression itself has been linked to various structural or functional changes of the brain. A systematic review and meta-analysis of structural MRI studies in patients with late-life depression revealed gray matter volume reduction over hippocampus, orbitofrontal cortex, putamen, and thalamus (Sexton et al., 2013). Another meta-analysis focused on resting-state functional connectivity studies among patients with MDD revealed large-scale network dysfunction, including hypo-connectivity within the frontoparietal network, hypo-connectivity between frontoparietal systems and parietal regions of the dorsal attention network, hypo-connectivity between emotion or salience network and midline cortical regions with top-down regulation functions, hyper-connectivity within the default network, and hyper-connectivity between frontoparietal control systems and regions of the default mode network (Kaiser et al., 2015). In the contrary, only limited structural or functional brain imaging studies focused on subjects with depressive symptoms. Gray matter volume deficits were found over frontal cortex, anterior cingulate cortex, and temporal regions (Taki et al., 2005; Dotson et al., 2009b; Webb et al., 2014). Increased functional connectivity between dorsal anterior cingulate cortex and dorsolateral prefrontal cortex (DLPFC; Li et al., 2014), and between precuneus and left orbitofrontal cortex (Felder et al., 2012) were demonstrated.

Few imaging studies have focused on loneliness-associated brain changes. In a voxel-based morphometry (VBM) study conducted among 108 healthy adults, lonely individuals had decreased gray matter in left posterior superior temporal sulcus, which is an area related to basic social perception (Kanai et al., 2012). Another VBM study revealed that lonely individuals had greater regional gray matter volume in left DLPFC and postulated that this finding reflected immature emotional regulation (Kong et al., 2015). In an fMRI study conducted among 23 healthy undergraduate students, a difference in brain activation pattern between lonely and non-lonely subjects when performing social perception tasks was demonstrated (Cacioppo et al., 2009). Lonely individuals exhibited a weaker activation of the ventral striatum to pleasant pictures of people than of objects and a greater activation of the visual cortex to unpleasant pictures of people than of objects. An fMRI study conducted among 36 schizophrenia patients and 40 normal controls has revealed that the increased insula activation toward covert facial expressions of disgust in schizophrenia patients was positively correlated with social loneliness (Lindner et al., 2014).

From these previous studies, alterations in brain regions related with functions of social perception, reward processing, and emotion regulation have been associated with loneliness (Cacioppo et al., 2009; Kanai et al., 2012; Lindner et al., 2014; Kong et al., 2015). However, except the study performed by Lindner et al. (2014) none of these studies had controled the effect of depression on brain alterations.

Further studies are needed for a more comprehensive understanding of the effects exerted on human brain by depressive symptoms and loneliness, especially since depressive symptoms and loneliness are highly correlated. This study aims to search for brain areas with functional connectivity density (FCD; Tomasi and Volkow, 2010) changes associated with either depressive symptoms or loneliness in a group of non-demented male elders by conducting a resting state fMRI analysis.

## Materials and Methods

### Participants

This study cohort comprised 85 Han Chinese male participants over 65 years of age recruited from a Veteran’s Home in northern Taiwan. Each participant was evaluated by a trained research assistant with Mini-International Neuropsychiatric Interview to exclude the presence of Axis I psychiatric disorders (Sheehan et al., 1998). In addition, all participants were assessed for cognitive function by Mini-Mental State Examination (MMSE; Folstein et al., 1975) Wechsler Digit Span Task (Wechsler, 1997), and Clinical Dementia Rating Scale (CDR; Hughes et al., 1982). Exclusion criteria for all participants consisted of the following: (1) presence of dementia (i.e., CDR > 0); (2) presence of Axis I psychiatric disorders, such as schizophrenia, bipolar disorders, or unipolar depression; and (3) a history of neurological conditions, such as head injury, stroke, or Parkinson’s disease. None of the participants had any contraindications to MRI scans.

This study received approval from the Institutional Review Board at Taipei Veterans General Hospital and was conducted according to the Declaration of Helsinki; written informed consent was obtained from all included subjects.

### Measurement of Depressive Symptoms and Loneliness

Depressive symptoms among all participants were assessed by the Chinese version of the Geriatric Depression Scale-short form (GDS; Lee et al., 1993) which contains 15 items. Each item was a yes/no question and a score of 0 or 1 could be assigned. The total score of GDS ranged from 0–15. A GDS score of 0–5 is normal and a score greater than five is suggestive of but not diagnostic for depressive disorder.

Severity of loneliness of each participant was assessed by the Loneliness Scale (University of California, Los Angeles, UCLA version 3; Russell, 1996) which contains 20 items. Participants used a 4-point Likert scale (ranging from “never” to “often”) to assess how often they felt the way described in the loneliness items. The severity of loneliness is higher with increased score of the Loneliness Scale but there is no cut point that indicates someone is lonely or not.

### Image Acquisition

fMRI scanning was performed at National Yang-Ming University using a 3.0 T Siemens MRI scanner (Siemens Magnetom Tim Trio, Erlangen, Germany) equipped with a 12-channel head coil. The scanning protocol was consistent with our prior reports (Yang et al., 2013, 2014). For resting-state image scanning, T2*-weighted images with BOLD contrast were measured using a gradient echo-planar imaging (EPI) sequence (repetition time TR = 2500 ms, echo time TE = 27 ms, FoV = 200 mm, flip angle = 77°, matrix size = 64 × 64, voxel size = 3.44 mm × 3.44 mm × 3.40 mm). For each run, 200 EPI volume images were acquired along the AC–PC plane. During resting state fMRI scans, all participants were instructed to keep their eyes closed, to remain as motionless as possible, to think of nothing specific, and to not fall asleep. After the resting scan, the technician asked the participants whether they fell asleep during the session, and the participants were rescanned if they slept during the resting scan. For structural image scanning, high-resolution structural T1 images were acquired with three-dimensional (3D) magnetization-prepared rapid gradient-echo sequence (3D-MPRAGE; TR = 2530 ms, TE = 3.5 ms, TI = 1100 ms, FoV = 256 mm, and flip angle = 7°). The whole MRI scanning session lasted about 15 min for each participant.

### Imaging Processing

Structural and resting image data were preprocessed and analyzed using SPM8 (Wellcome Department of Imaging Neuroscience, London, UK) implemented in MATLAB (Mathworks Inc., Sherborn, MA, USA). Structural T1 images were segmented and co-registered with resting functional images. Resting BOLD data were slice-timing corrected, re-aligned, and normalized into the standard stereotaxic space of Montreal Neurological Institute (MNI) EPI template, and resampled to a 3 mm cubic voxel. Covariates of BOLD time series were regressed out before performing FCD analysis, including the time courses of six head motions, white matter, and cerebrospinal fluid. No global signal regression was performed to avoid introducing distortions in the time series data (Murphy et al., 2009; Anderson et al., 2011). All participants included in this study exhibited a maximum displacement of less than 1.5 mm at each axis and an angular motion of less than 1.5° for each axis. The first five data points (12.5 s) in any BOLD time series were discarded because of the instability of initial MRI scanning, leaving 195 data points in final data. Temporal low-pass filtering (0.01–0.08 Hz) was performed to reduce the influence of high-frequency noise from physiologic confounders.

### Functional Connectivity Density Mapping

FCD mapping (Tomasi and Volkow, 2010, 2012) was developed based on resting-state BOLD signals for mapping whole-brain short-range and long-range functional connectivity with high spatial resolution (3-mm isotropic), which allows identification of functional hubs (Tomasi and Volkow, 2010, 2011). Briefly, Tomasi et al. defined three types of FCD measures, including global FCD (GFCD), short-range FCD (SFCD), and long-range FCD (LFCD).

For each gray matter voxel, GFCD was defined as the number of global functional connections, k(x_i_), determined through Pearson correlations between BOLD signals at voxel x_i_ and those in the remaining gray matter voxels using an arbitrary threshold, *r* > 0.6 (Tomasi and Volkow, 2010). SFCD was defined as local neighbors of voxel x_i_, which was computed using a growth algorithm to identify voxels that were adjacent to a voxel linked to x_i_ on a continuous path, and had connectivity strength of *r* > 0.6 with the voxel x_i_. This growth algorithm was repeated in an iterative manner until no new neighbors could be found.

In the last step, the LFCD of voxel x_i_ was computed by subtraction of GFCD and SFCD. Calculation of FCD was computed for all gray matter voxels. Whole brain short- and LFCD maps were further normalized by the whole-brain mean FCD, and were spatially smoothed with an 8-mm Gaussian kernel in SPM8 to minimize differences in brain functional anatomy across subjects (Tomasi and Volkow, 2012).

### Statistical Analysis

Statistical analyses of parametric imaging data were conducted using MATLAB. Regional correlations in whole-brain parametric mapping between depressive symptoms or loneliness and LFCD or SFCD were examined using general linear model (GLM). In the GLM analysis, age was incorporated as a covariate and depressive symptoms and loneliness were used as predictors of long-range or SFCD metrics. Significant brain clusters were reported if uncorrected *p* value was less than 0.001 for any *t*-test on a single voxel level with a cluster size greater than 30 voxels and a family-wise error (FWE)-corrected *p*-value <0.05.

## Results

### Demographic and Scale Scores

Demographic characteristics of the study participants were shown in Table 1. This cohort was comprised of 85 elderly male with a mean age of 80.3 ± 5.6 years. The mean duration of education was 5.7 ± 5.2 years. The average score of GDS was 1.61 ± 2.4 (range = 0–12). The percentage of patients with a GDS score smaller or equal to five was 91.8%. Although seven of the subjects had a GDS score greater than 5, they had no clinical diagnoses of depressive disorders. Regarding the severity of loneliness, the average score of the Loneliness Scale was 29.6 ± 8.7 (range = 20–61). GDS and loneliness were significantly correlated among our subjects (Pearson correlation coefficient = 0.523, *p* <0.001).

**Table 1:** **Demographic characteristics of study participants (*N* = 85)**.

Variables
Age, year	80.3 ± 5.6
Sex, male	85 (100.0)
Handedness, right	83 (97.6)
Education, year	5.7 ± 5.2
Geriatric depression scale	1.6 ± 2.4
Loneliness scale	29.6 ± 8.7
Mini mental state examination	26.6 ± 2.8
	(*N* = 84)

*Categorical data are given as number (%)*.

### Functional Connectivity Density Changes Changes

**Figure 1:**
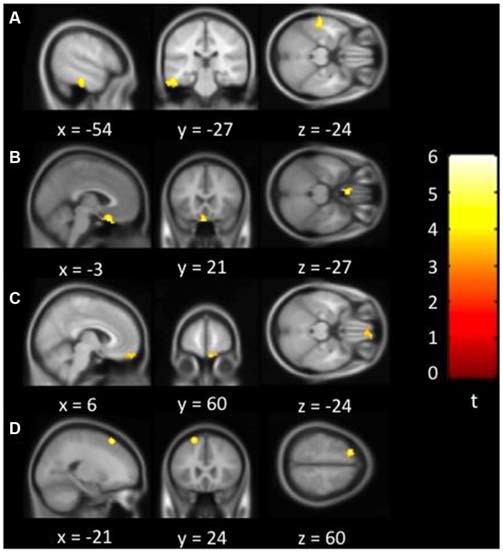
**Brain regions showing positive correlation between long-range functional connectivity density (FCD) and depressive symptoms. (A)** Left inferior temporal gyrus. **(B)** Left rectal gyrus. **(C)** Right supraorbital gyrus. **(D)** Left superior frontal gyrus.

**Figure 2:**
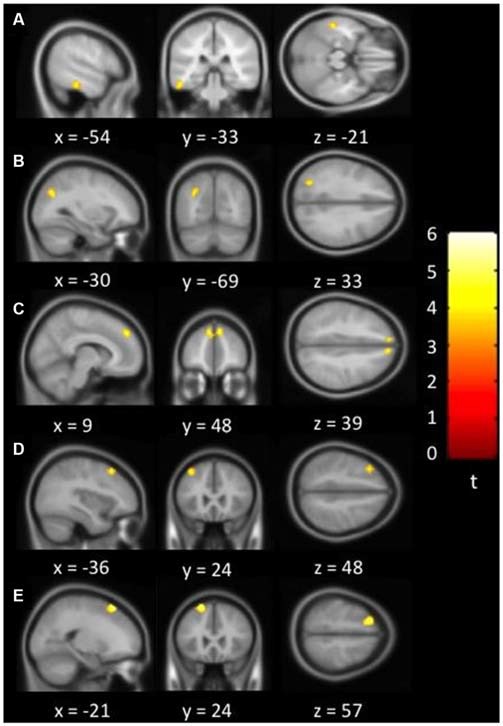
**Brain regions showing positive correlation between short-range FCD and depressive symptoms. (A)** Left inferior temporal gyrus. **(B)** Left middle occipital region. **(C)** Bilateral superior medial frontal gyrus. **(D)** Left middle frontal gyrus. **(E)** Left superior frontal gyrus.

Figure 1 showed regions with significant correlation between LFCD and depressive symptoms after controlling for age and loneliness. There were four clusters that demonstrated positive association between LFCD and GDS, including left inferior temporal gyrus (*t* = 4.57), left rectal gyrus (*t* = 4.48), right supraorbital gyrus (*t* = 3.87), and left superior frontal gyrus (*t* = 4.46) (Table 2). Figure 2 showed regions with significant correlation between SFCD and GDS after controlling for age and loneliness. There were five clusters that demonstrated positive association between SFCD and GDS, including left inferior temporal gyrus (*t* = 4.18), left middle occipital region (*t* = 4.11), bilateral superior medial frontal gyrus (*t* = 4.32), left middle frontal gyrus (*t* = 3.92), and left superior frontal gyrus (*t* = 4.82) (Table 2). There were no regions exhibiting negative correlation between LFCD or SFCD and GDS.

**Table 2:** **Brain regions showing a significant correlation between functional connectivity density (FCD) and depressive symptoms**.

Brain region^a^	BA	MNI coordinates (mm)			Volume (mm^3^)^b^
		*x*	*y*	*z*		Peak *t*	Effect size *r*
**Long-range FCD**
L inferior temporal gyrus	20	−54	−27	−24	3942	4.57	0.45
L rectal gyrus	11	−3	21	−27	1404	4.48	0.44
R supraorbital gyrus	11	6	60	−24	1593	3.87	0.39
L superior frontal gyrus	6	−21	24	60	1080	4.46	0.44
**Short-range FCD**
L inferior temporal gyrus	20	−54	−33	−21	1080	4.18	0.42
L middle occipital	19	−30	−69	33	837	4.11	0.41
L/R superior medial frontal	9	9	48	39	1701	4.32	0.43
L middle frontal	8	−36	24	48	837	3.92	0.40
L superior frontal	8	−21	24	57	1674	4.82	0.47

*^a^L, left; R, right; BA, Brodmann area; ^b^Volume was computed from cluster size (3 × 3 × 3 mm voxel). All brain clusters had uncorrected p value <0.001*.

**Figure 3:**
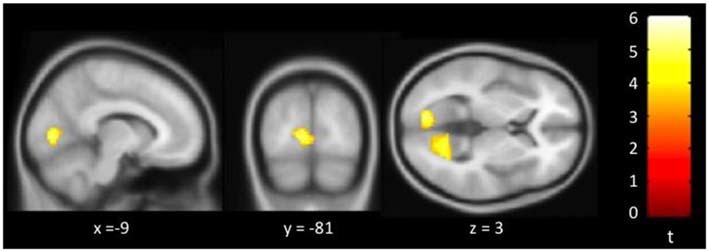
**Brain regions showing positive correlation between short-range functional connectivity density (SFCD) and loneliness**. Bilateral lingual gyrus.

Figure 3 showed regions with significant correlation between SFCD and loneliness after controlling for age and GDS. There was one cluster which demonstrated positive association between SFCD and loneliness, over bilateral lingual gyrus (*t* = 5.10) (Table 3). No regions exhibiting positive or negative correlations between LFCD and loneliness were found.

**Table 3:** **Brain regions showing a significant correlation between FCD and loneliness**.

		MNI coordinates (mm)		
Brain region^a^	BA	*x*	*y*	*z*	Peak *t*	Volume (mm^3^)^b^	Effect size *r*
**Short-range FCD**
L/R lingual gyrus	18	−9	−81	3	7506	5.10	0.49

*^a^L, left; R, right; BA, Brodmann area; ^b^Volume was computed from cluster size (3 × 3 × 3 mm voxel). All brain clusters had uncorrected p value <0.001*.

## Discussion

Our study aimed to search for brain regions with FCD changes associated with depressive symptoms or loneliness in non-demented male elders. We found that FCD changes were associated with both depressive symptoms and loneliness in these subjects. The brain regions of FCD changes associated with depressive symptoms after controlling for age and loneliness were located mostly in frontal and temporal regions. Significant positive correlations between depressive symptoms and LFCD were observed in left rectal gyrus, left superior frontal gyrus, right supraorbital gyrus, and left inferior temporal gyrus. Positive associations between depressive symptoms and SFCD were observed in left middle frontal gyrus, left superior frontal gyrus, bilateral superior medial frontal gyrus, left inferior temporal gyrus, and left middle occipital region. In addition, positive association between SFCD and loneliness was centered over bilateral lingual gyrus. At present, no other study has investigated brain FCD changes associated with depressive symptoms or loneliness.

### Associations of Depressive Symptoms and Functional Connectivity Density

Due to inadequacy of current literature regarding the associations of depressive symptoms and FCD changes, we compare our results to contemporary findings in three different perspectives: (1) FCD studies conducted among patients with MDD; (2) brain imaging studies conducted in subjects with depressive symptoms; and (3) brain imaging studies among subjects with clinical depressive disorders.

Only one study focused on FCD changes among patients with MDD (Zhang et al., 2015). This study revealed reduced GFCD in mid-cingulate cortex and increased GFCD in occipital cortex in MDD patients. In addition, they did not find significant correlations between Hamilton depression scores and GFCD values. In terms of the outcome measures, they used GFCD as opposed to SFCD and LFCD used in our study that may in part explain the difference between their results and ours and the lack of association between depressive symptoms and FCD changes in their study may be associated with a relatively small sample size of 21 MDD patients.

The subjects in our study were a group of male non-demented elders with variable depressive symptoms as measured by GDS but did not warrant the diagnosis of clinical depressive disorders. Few brain imaging studies have focused on subjects with depressive symptoms. A VBM study revealed reduced gray matter volumes over superior frontal gyrus, orbitofrontal cortex, anterior cingulate cortex, thalamus, superior temporal gyrus, and temporal pole in subjects with depressive symptoms (Webb et al., 2014). Another study focused on elderly population with depressive symptoms showed reduced gray matter volumes over orbitofrontal cortex, cingulate gyrus, and left temporal lobe (Dotson et al., 2009b). Reduced gray matter volumes over bilateral superior medial frontal areas were also demonstrated in a group of male elders with subthreshold depression (Taki et al., 2005). Various alterations associated with depressive symptoms were also shown in functional brain imaging studies. A positron emission tomography study showed reduced regional cerebral blood flow in frontal and temporal regions in elderly subjects with chronic depressive symptoms (Dotson et al., 2009a). Increased functional connectivity was observed between dorsal anterior cingulate cortex and DLPFC (Li et al., 2014), and between precuneus and left orbitofrontal cortex (Felder et al., 2012). These findings were consistent with our findings that brain regions associated with depressive symptoms in male non-demented elders were mainly over frontal and temporal regions.

Further comparing our findings to previous literatures focusing on brain alterations with clinical depressive disorders, overlapping regions over frontal and temporal regions were also demonstrated. Firstly, left superior and middle frontal gyri, which were part of left DLPFC, showed a positive correlation between GDS and SFCD in our study. In an MRI functional connectivity study comparing MDD patients and healthy controls, MDD patients demonstrated increased functional connectivity between left DLPFC and part of frontal gyrus, cingulate cortex, and parietal cortex (Shen et al., 2015). In another resting state functional connectivity study, increased connectivity was shown between left DLPFC and pregenual anterior cingulate cortex among MDD patients. Secondly, our study also showed a positive correlation between GDS and LFCD over left rectal gyrus and right supraorbital gyrus, which were part of the orbitofrontal cortex. Greater resting-state functional connectivity between dorsomedial insular cortex and amygdala, subgenual prefrontal cortex, orbitofrontal cortex were demonstrated in a study comparing MDD patients and normal controls, and increased functional connectivity was positively correlated with depression severity (Avery et al., 2014). Thirdly, in our study, positive correlations between GDS and both SFCD and LFCD were noted over left inferior temporal gyrus; positive correlation between GDS and SFCD was observed among bilateral superior medial frontal gyrus. In a study focusing on late-life depression patients, increased resting-state functional connectivity was demonstrated between dorsomedial prefrontal cortex and orbitofrontal cortex (Wu et al., 2011). Another study revealed decreased resting state functional connectivity between cerebellum and multiple frontal, parietal and temporal regions including the inferior temporal gyrus (Guo et al., 2013).

Taken together, previous studies focusing on resting-state functional brain MRI in subjects with depressive disorders revealed alterations mainly involving three neural functional networks, namely the cognitive control network (mainly containing DLPFC), the affective network (comprising amygdala, insula, globus pallidum, orbitofrontal cortex, and temporal poles), and the default mode network (including medial prefrontal cortex, posterior cingulate cortex, inferior temporal gyrus, and inferior parietal lobule; Greicius et al., 2007; Veer et al., 2010; Alexopoulos et al., 2012). Our findings indicate that brain regions demonstrating positive correlations between depressive symptoms and FCD changes may involve the three main networks with alterations in patients with depressive disorders, with left superior and middle frontal gyrus included in the cognitive control network, left rectal gyrus and right supraorbital gyrus included in the affective network, and left inferior temporal gyrus and bilateral medial prefrontal cortex included in the default mode network.

Of note, in contrast to both increased and decreased functional connectivity strength in patients with depression or depressive symptoms demonstrated in previous literature, our study only revealed positive correlations between depressive symptoms and long- or SFCD and there were no significant negative correlations observed. This may result from the inherent difference between the definitions of FCD and functional connectivity strength, in that functional density of one voxel may be viewed as, although not tantamount to, the combination of all its one-to-one functional connectivity strengths to all the other voxels in the brain. Although it is still inconclusive whether increased FCD represents a deleterious change or a compensatory effect associated with depressive symptoms, our findings of FCD changes implicated the importance of network-level brain alterations in subjects with depressive symptoms.

### Associations of Loneliness and Functional Connectivity Density

Previous studies have demonstrated that loneliness was associated with social cognition abnormalities such as hypervigilance for social threats (Qualter et al., 2013) and aberrations in decoding social cues (Gardner et al., 2005). In our study, positive correlations between SFCD and loneliness were observed over bilateral lingual gyrus. Lingual gyrus and middle occipital regions are associated with visual processing, self-reference, and social perception. A VBM study delineated the relationship between perceived social support and gray matter volume changes (Che et al., 2014). They showed that among healthy subjects, perceived social support was positively associated with the volume over bilateral lingual cortex, posterior parts of posterior cingulate cortex, left occipital lobe and cuneus. Another VBM study conducted among patients with social anxiety disorder revealed that gray matter volume over lingual gyrus was increased in patients and its volume changes were positively associated with symptom severity (Frick et al., 2014). An fMRI study also showed that the activity in fusiform gyrus, superior temporal gyrus and lingual gyrus were associated with perception of social dominance from facial postures (Chiao et al., 2008). Lingual gyrus is implicated as an important brain region involving in social cognition and processing in these studies. Our study results suggested that the effect of loneliness on brain FCD changes might be predominantly over brain regions associated with social cognition, which were in line with the concept that perceived social isolation was an important factor in loneliness.

From our current study, both depressive symptoms and loneliness were demonstrated to be associated with FCD changes in various brain regions. Further longitudinal studies are warranted to investigate possible underlying causal relationships. While at the same time, interventions for elderly population with depressive symptoms and loneliness may be necessary in order to reduce their detrimental effects on physical and mental health.

### Strengths and Limitations

To the best of our knowledge, this is the first study to investigate brain regions with resting-state functional connectivity changes associated with loneliness. Implementing FCD mapping is one of the strengths of the current study; compared to seed-based functional connectivity study, voxel-wise FCD mapping is not biased by predetermined region of interest. There are also some important limitations in our study. Firstly, depressive symptoms and loneliness were correlated with each other. We employed GLM analysis to examine the independent effect of depressive symptoms or loneliness on FCD changes, and may provide a better understanding of the individual associations between depressive symptoms or loneliness and FCD changes. Secondly, because this is a cross-sectional study, we should be careful not to interpret the associations between either depressive symptoms or loneliness and regional FCD changes of the brain as a causal effect. Finally, all subjects in the study were male, therefore the results could not be extrapolated into female subjects.

## Conclusion

FCD changes were associated with depressive symptoms and loneliness in a group of non-demented male elders. Depressive symptoms mainly exhibit a positive correlation with FCD changes over frontal and temporal regions, which may include regions of the cognitive control network, affective network, and default mode network. Loneliness exhibits a positive correlation with FCD changes over bilateral lingual gyri that are important in social cognition. These results suggest that depressive symptoms and loneliness may be associated with different brain regions in non-demented elderly male.

## Conflict of Interest Statement

The authors declare that the research was conducted in the absence of any commercial or financial relationships that could be construed as a potential conflict of interest.
